# Polygenic loading for major depression is associated with specific medical comorbidity

**DOI:** 10.1038/tp.2017.201

**Published:** 2017-09-19

**Authors:** T H McCoy, V M Castro, L Snapper, K Hart, J L Januzzi, J C Huffman, R H Perlis

**Affiliations:** 1 Center for Quantitative Health, Center for Human Genetic Research and Department of Psychiatry, Massachusetts General Hospital, Boston, MA, USA; 2 Partners Research Information Systems and Computing, Partners HealthCare System, One Constitution Center, Boston, MA, USA; 3 Cardiology Division, Massachusetts General Hospital and Harvard Clinical Research Institute, Boston, MA, USA; 4 Department of Psychiatry, Massachusetts General Hospital, Boston, MA, USA

## Abstract

Major depressive disorder frequently co-occurs with medical disorders, raising the possibility of shared genetic liability. Recent identification of 15 novel genetic loci associated with depression allows direct investigation of this question. In cohorts of individuals participating in biobanks at two academic medical centers, we calculated polygenic loading for risk loci reported to be associated with depression. We then examined the association between such loading and 50 groups of clinical diagnoses, or topics, drawn from these patients' electronic health records, determined using a novel application of latent Dirichilet allocation. Three topics showed experiment-wide association with the depression liability score; these included diagnostic groups representing greater prevalence of mood and anxiety disorders, greater prevalence of cardiac ischemia, and a decreased prevalence of heart failure. The latter two associations persisted even among individuals with no mood disorder diagnosis. This application of a novel method for grouping related diagnoses in biobanks indicate shared genetic risk for depression and cardiac disease, with a pattern suggesting greater ischemic risk and diminished heart failure risk.

## Introduction

Major depression has been associated with a host of non-psychiatric comorbidities, ranging from cardiovascular disease to autoimmune disorders. The impact of co-occurrence is often profound and bi-directional—that is, outcomes of each disorder tend to be worse in the presence of the other,^1, 2, 3, 4, 5^ despite the availability of multiple efficacious treatments.^6^ Thus, understanding the mechanism of such co-occurrence has both scientific and clinical relevance.

This co-occurrence has a range of possible explanations. It is possible that depression increases risk for some disorders, either directly (for example, via changes in cortisol or immunosuppression) or indirectly (for example, via changes in health behaviors).^7, 8^ Conversely, the presence of a medical disorder can represent a stressor increasing risk for depression.^9^ Yet, a third model posits a shared liability—i.e., the same factors that increase risk for one disorder may increase risk for the other.^10^ Multiple twin or family investigations support this shared liability, at least for cardiovascular disease.^11, 12, 13^ In reality, it is likely that all three mechanisms play a role in co-occurrence of some disorders, but confirming the presence of at least one mechanism would represent a key first step in understanding these relationships.^14^

Multiple novel genetic loci associated with major depression in individuals of Northern European ancestry at a genome-wide level of significance have recently been reported.^15^ These newly reported loci allow for direct examination of the possibility that aggregated genetic liability for depression, in addition to depression itself, is associated with risk for disorders other than depression. The present study aims to test the association between such liability and medical comorbidity, using a novel method for deriving this comorbidity. Specifically, we drew on these newly reported genetic associations to construct estimates of common-variant genetic loading in the large medical biobanks from two academic medical centers. We then tested these depression loading measures for association with empirically derived groups of diagnoses, or topics, that tend to co-occur with each other.

## Materials and methods

### Clinical phenotype derivation

Standard phenome-wide association studies (PheWAS) test all diagnostic codes against all predictors—typically individual risk variants or genome-wide common variants.^16^ This approach risks either inflation of type I error (by testing 1500+ diagnostic codes), or type II error (by correcting for 1500+ diagnostic codes). Further, it fails to take into account correlation between individual coded diagnoses, and the highly variable reliability of many such codes.

**Figure 1:**
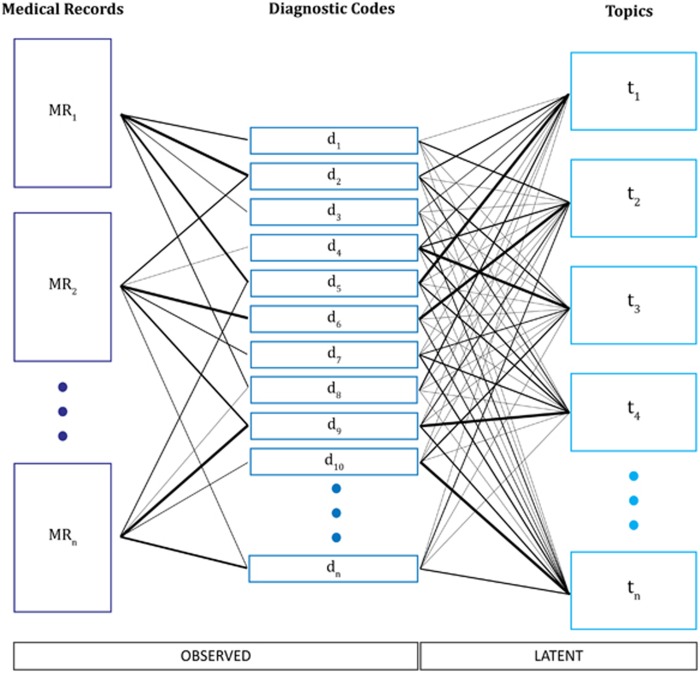
Illustration of the process of topic modeling as applied in this study.

As an alternative, we developed a method that applies latent Dirichilet allocation (LDA) to reduce categorical diagnostic ontologies to a finite set of topics on which these codes load.^17, 18^ This form of unsupervised machine learning has most commonly been applied in natural language processing to capture the topics expressed in documents; it presumes that individual tokens or terms (here, diagnostic codes) reflect an underlying topic, and that the record of an individual patient reflects a combination of latent topics. Figure 1 depicts the process of conceptualizing patient medical records as a 'bag' of observed diagnostic code counts from which unobserved latent topics are inferred using LDA. Thereafter, the inferred topics are treated as the phenotype in analysis.

Here we extracted all ICD-9 diagnostic codes for biobank participants from the inpatient and outpatient electronic medical records of Massachusetts General Hospital and Brigham and Women's Hospital and grouped them into 1667 PheWAS disease categories.^19^ All participants had signed written informed consent for biobank participation, including consent for release of deidentified data under a Data Use Agreement to qualified investigators, as approved by the hospitals' Institutional Review Board. We eliminated PheWAS codes occurring in <1% of individuals or more than 99% of individuals in the first cohort, leaving 508 codes for topic model construction. We then trained a 50-topic model, and scored cohorts one and two. (The decision a priori to select 50 topics is discussed further below; the optimal number of targets remains an area of research in unsupervised learning^20, 21, 22, 23^). The LDA was performed using the Gensim implementation.^24, 25^ Importantly LDA allows for the possibility of all codes with respect to all topics. The distinction between topics is in the expected probabilities of each code. As such, we focus on the most probable diagnostic codes given each topic as a means of interpreting the topics. When individual topics are mentioned in the text they are named using the most strongly loaded code with the suffix ‘++’ to indicate that a topic comprises many codes, each contributing to membership in that topic.

### Molecular methods

All subjects were genotyped using either the Illumina MEGA (cohort 1, *n*=4931) or the Illumina MEGA-EX (cohort 2, *n*=4428) array (Illumina; San Diego, CA, USA). Each cohort was cleaned, imputed, and analyzed separately to minimize batch effects. We retained subjects with genotyping call rates exceeding 99% and no evidence of relatedness based on identity by descent (IBD).^26^ We likewise retained any SNPs with call rate of 95% or greater, and Hardy–Weinberg equilibrium *P*-value>1 × 10^−10^. Genotypes were next imputed using the Michigan Imputation Server implementing Minimac3, based on all population subsets from 1000 Genomes Phase 3 v5 as reference panel.^27, 28, 29^ Phasing of haplotypes used SHAPEIT.^30^

We generated principal components, as implemented in PLINK 1.9, to identify the first 10 components of the variance-standardized relationship matrix among genotyped SNPs in each cohort.^31^ After overlaying HapMap populations, only those individuals falling within the Northern European cluster were included in subsequent analysis.

### Analysis

We generated polygenic risk scores (PRS), estimates of polygenic loading for MDD, using seven tranches of SNPs (5 × 10^−8^, 1 × 10^−7^, 1 × 10^−6^, 1 × 10^−5^, 1 × 10^−4^, 1 × 10^−3^ and 1 × 10^−2^, S1–S7) drawn from our prior publication reporting 15 genome-wide associations with depression.^15^ The value for each *P*-value tranche represents the maximum *P*-value that is included in that tranche. This list was linkage-disequilibrium pruned using the 'clump' function as implemented in PLINK 1.9, with a 250 kb window and minimum r2 set at 0.5 by default.^32^

We used linear regression to examine association between depression polygenic score and each of the 50 topics. We fit both unadjusted models and models incorporating the first 10 MDS components, and present the meta-analyzed result of the two genotyping cohorts.

As this analysis was intended as a hypothesis-generating effort, we present uncorrected *P*-values in all results. For purposes of interpretation, Bonferroni correction for 50 topics would require a *P*-value of 0.05/50, or 0.001, for significance. The seven PRS tranches are correlated so do not represent seven independent tests per phenotype; since the average r between them is ~0.65, we consider a fully corrected *P*-value threshold for significance to be 0.00042.^33, 34^ To further elucidate the statistical significance of any associations identified we utilized permutation to calculate empirical *P*-values as well as an experiment-wide *P*-value. To do so, we randomized the relationship between the topics (phenotype) and the MDS adjusted PRS (genotype) and calculated association in the full cohort between all tranches and all topics 100 000 times under this simulated null.

## Results

In the first genotyping cohort, there were 3728 individuals, including 2165 females (58.1%) and 997 individuals (26.7%) with a mood disorder. Cohort 2 included 2712 individuals, including 712 (49.2%) females and 779 individuals (28.7%) with a mood disorder. Mean age in cohort 1 was 57.6 (s.d. 16.8) years; mean age in cohort 2 was 62.2 (s.d. 15.9) years.

**Figure 2:**
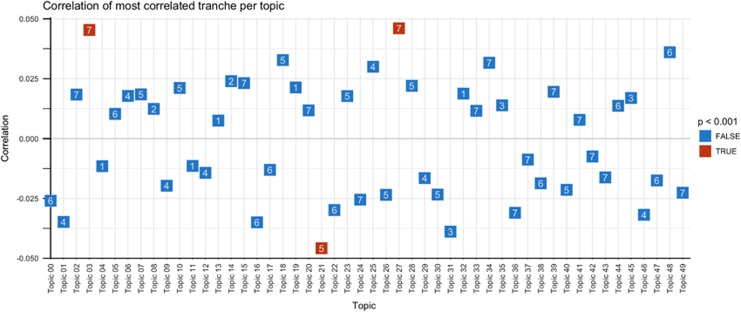
Distribution of associations of polygenic risk scores (PRS) by topic (*x* axis) and minimum *P*-value across seven PRS tranches (*y*axis).

Figure 2 illustrates the distribution of associations with PRS by topic and minimum *P*-value (that is, PRS threshold yielding strongest evidence of association). For consistency with other data clustering methods, topics are named according to predominant terms, adding the suffix ‘++’ to indicate that topics may contain overlapping terms as well as apparently unrelated terms. Three topics—mood disorder++ (03), heart failure++ (21) and cardiac ischemia++ (27)—met an experiment-wide threshold for association (Table 1). PheWAS diagnostic codes contributing to these topics are listed in Table 2, ranked by order of contribution (that is, weighting of each code for a given topic, from greatest to least). (Supplementary Table 1 reports all topics by all PRS tranches, sorted by association *P*-value). Permuted topic level associations matched those of the primary analysis (Supplementary Figure 1). In experiment-wide permutation analysis the number of significant topics was itself statistically significant (permuted *P*=0.02).

**Figure 3:**
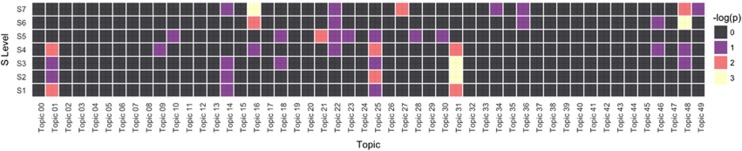
Heat map of association for all topics, by polygenic risk scores (PRS) tranche excluding depression cases.

In secondary analysis, we excluded any individuals with a mood disorder PheWAS code (*n*=1776) and repeated these analyses. Association *P*-values are indicated in Table 1 (right), and visualized in a heat map in Figure 3. As anticipated, the mood disorder topic (03) was no longer significant, suggesting that the additional codes in that topic do not contribute meaningfully to association; the two cardiac topics demonstrate persistent association.

## Discussion

In this analysis of electronic health record data from 6440 individuals of Northern European ancestry, we identified three sets of diagnoses (topics) associated with PRS for depression at an experiment-wide significance threshold. One of these encompasses mood disorders, and can be considered a positive control or indicator of assay sensitivity—though notably, it also includes related codes (adjustment disorder, tobacco use, and anxiety) that may reflect true pleiotropy or simply differences in the way mood and anxiety symptoms are coded. The other two reflect different elements of cardiac pathology. That cardiac pathology surfaces from this unsupervised machine learning approach is reassuringly face valid given the extensive literature relating cardiac and mood disorders; however, cardiac pathology is subdivided into distinct associations.^1, 2, 3, 4^ Cardiac ischemia++ (topic 27) largely captures acute coronary syndromes, including risk factors for such syndromes (See Table 2 for the codes most strongly associated with this topic). The data suggest that MDD loading is associated with greater risk for these acute syndromes (Table 1). Heart failure++ (topic 21) also reflects cardiovascular pathology but appears to reflect a more chronic disease course, including heart failure but not limited to cardiac disease (Table 2). Depressive genetic loading is inversely associated with heart failure++ (Table 1). This differential result underscores the complexity of the relationship between depression and cardiovascular disease.^35, 36, 37^ Importantly, evidence of association persists even when individuals with a mood disorder are excluded, suggesting that the observed associations are not simply consequences of a mood disorder diagnosis.

The relationship between depressive disorder and acute coronary syndromes is well described; however, the mechanism remains unclear.^38, 39, 40, 41, 42^ Multiple investigations using twin- and family-based designs have found evidence of shared heritable liability between MDD and cardiac disease.^11, 12, 13^ One study suggests the complexity of this relationship, with the extent of liability varying with sex and age.^13^ Further complicating efforts to understand comorbidity is the observation that depression is associated with increased deleterious behavior such as tobacco use and less exercise - notably tobacco use, for which risk is likely to be heritable, contributes to both a mood++ and a cardiovascular++ topic in our results. On the other hand, in prior studies individuals with major depression exhibit greater platelet reactivity and prevalent endothelial dysfunction relative to non-depressed subjects, which is a candidate pathway for depression increasing cardiac risk directly.^43^

Surprisingly, in the present study depression loading is inversely associated with more chronic forms of cardiac disease (heart failure++ topic 21). Although it is speculative, the divergences between acute and chronic cardiac pathology may reflect the tension between genetic and behavioral determinates of health. In the chronic phases of cardiac disease, personality is an important determinate of outcome.^44, 45, 46^ Alternatively, those with heart failure and comorbid depression might in fact have substantially higher mortality risk, obscuring a chronic link between the two diagnoses due to attrition.

In interpreting these associations, it is important to recognize that the methodology itself identifies co-occurring codes; the application of names to the underlying (that is, latent) concepts requires human intervention. Moreover, codes are associated, even if only negligibly, with all topics. This characteristic represents both a feature and a limitation of the methodology: it does not require manual curation of topics, and thus can discover relationships in data not specified a priori; on the other hand, it requires interpretation of results that may not be intuitive. Concretely, we would not have expected acute and chronic cardiac disease to primarily occur in distinct topics. This surprise bespeaks the hypothesis-generating potential of unsupervised machine learning, which still requires interpretation and follow-up. For ease of reading, by convention we name each topic by the most strongly-loading code, but again emphasize that a topic is not simply a single code and note that visualization and interpretation of probabilistic topics remain an area of active investigation in machine learning.^47^ Conversely, for testing specific hypotheses about a particular set of diagnoses, merely testing a curated code or set of codes would be most interpretable, but preclude discovery of new or unanticipated relationships.

Latent Dirichilet allocation has been applied extensively in natural language processing to identify topics reflected in blocks of text; however, to our knowledge, it has not been utilized for genetic investigation. The present study suggests the utility of this method for understanding the relationship between psychiatric and comorbid disorders, and we hope this study will prompt further investigations in larger biobanks and in using other polygenic risk measures. Still, an important caveat to this work is the inability to determine causation. Specifically, we cannot distinguish between the case of shared genetic liability for depression and comorbidities, and depression ‘causing’ the observed comorbidity. However, we are able to render the third possibility (the comorbidity causes the depression) less likely, by conditioning on an aggregate measure of genetic depression liability. A further caveat is the likelihood that other parameters would yield differing results—i.e., the application of 10 or 100 topics. Here, we selected 50 as a plausible number of disease groups *a priori*, and based on prior work with LDA in other contexts. Understanding the extent to which other, nonlinear means of weighting diagnoses, or specifying other numbers of topics, would yield differing results merits further study.

Taken together, our results suggest the complexity of the relationship between mood disorders and comorbid somatic illness, and indicate specific groups of diagnoses that may travel along with genetic risk for major depression. They further illustrate the application of a novel approach to aggregating diagnoses, applicable to any large clinical data set, which may be more tolerant of heterogeneity in diagnostic codes and sensitive to groups of diagnoses that travel together. At the same time, the observed association with mood disorders may be considered further replication of the previous report of depression liability genes.^15^ The application of topic modeling therefore appears to be a promising strategy.

## Figures and Tables

**Table 1:** Topics associated with PRS at an experiment-wide threshold for significance in primary analysis (left); follow-up analysis of primary associations (right)

*Topic*	P*-value tranche*	*Primary analysis*				*Excluding individuals with mood disorder diagnosis*			
		*Coefficient*	*95% CI*		*Association* P-*value*	*Coefficient*	*95% CI*		*Association* P-*value*
Topic 27: Ischemic Heart Disease++	1 × 10^−2^	0.046	0.022	0.070	0.000221	0.046	0.017	0.074	0.00185
Topic 21: Heart Failure++	1 × 10^−4^	−0.046	−0.070	−0.021	0.000237	−0.044	−0.072	−0.015	0.00279
Topic 03: Mood Disorders++	1 × 10^−2^	0.045	0.021	0.070	0.000273	−0.010	−0.039	0.018	0.47953

Abbreviations: CI, confidence interval; PRS, polygenic risk scores.

**Table 2:** List of top 20 PheWAS codes contributing to topics associated with PRS

*Topic 3: Mood disorders++*	*Topic 21: Heart failure++*	*Topic 27: Ischemic heart disease++*
Mood disorders	Heart failure	Ischemic Heart Disease
Anxiety phobic and dissociative disorders	Cardiomyopathy	Cardiac conduction disorders
Substance addiction and disorders	Ill-defined descriptions and complications of heart disease	Tobacco use disorder
Pervasive developmental disorders	Cardiac conduction disorders	Pulmonary collapse; interstitial/compensatory emphysema
Schizophrenia and other psychotic disorders	Cardiomegaly	Ill-defined descriptions and complications of heart disease
Adjustment reaction	Ischemic Heart Disease	Hyperplasia of prostate
Tobacco use disorder	Pulmonary congestion and hypostasis	Congenital musculoskeletal anomalies
Back pain	Renal failure	Hypotension
Malaise and fatigue	Pleurisy; pleural effusion	Cardiomegaly
Other headache syndromes	Pulmonary collapse; interstitial/compensatory emphysema	Vertiginous syndromes and other disorders of vestibular system
Sleep disorders	Other forms of chronic heart disease	Pleurisy; pleural effusion
Abdominal pain	Heart valve disorders	Syncope and collapse
Acute upper respiratory infections	Disorders of fluid electrolyte and acid-base balance	Symptoms/disorders of the urinary system
Superficial cellulitis and abscess	Other anemias	Benign neoplasm of colon
Constipation	Hypotension	Overweight
Alcohol-related disorders	Diabetes mellitus	Sleep disorders
Disorders of sweat glands	Pneumonia	Malaise and fatigue
Other nutritional deficiency	Shock	Varicose veins
Neurological disorders due to brain damage	Symptoms involving skin and other integumentary tissue	Other dermatoses
Delirium dementia and amnestic disorders	Syncope and collapse	Heart valve disorders

Abbreviation: PRS, polygenic risk scores.
