# Supplementary figures and images for: Polygenic loading for major depression is associated with specific medical comorbidity
Source: Transl Psychiatry. 2017 Sep 19;7(9):e1238–. doi: 10.1038/tp.2017.201 (PMC5639245; doi:10.1038/tp.2017.201)

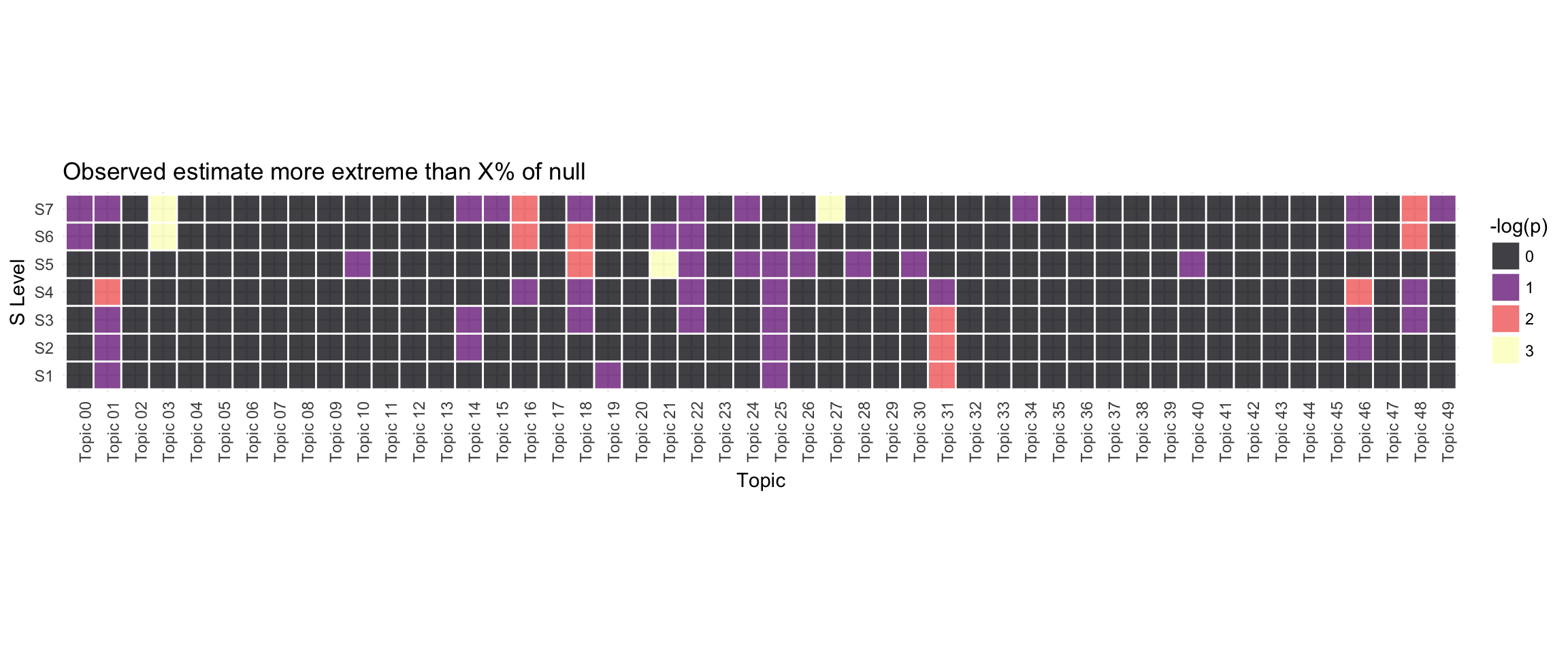

Supplement: Supplementary Figure 1 [file tp2017201x2.png]
